# Editorial: Neuro-Immune Connections to Enable Repair in CNS Disorders

**DOI:** 10.3389/fimmu.2020.01425

**Published:** 2020-07-24

**Authors:** Tim Vanmierlo, Jack van Horssen, Niels Hellings, Bieke Broux

**Affiliations:** ^1^Neuro-Immune Connections and Repair Lab, Department of Immunology and Infection, Biomedical Research Institute, University MS Center, Hasselt University, Hasselt, Belgium; ^2^Division Translational Neuroscience, School for Mental Health and Neuroscience, Maastricht University, Maastricht, Netherlands; ^3^Department of Molecular Cell Biology and Immunology, MS Center Amsterdam, Amsterdam Neuroscience, Amsterdam UMC, Vrije Universiteit Amsterdam, Amsterdam, Netherlands; ^4^Department of Internal Medicine, Cardiovascular Research Institute Maastricht, Maastricht University, Maastricht, Netherlands

**Keywords:** neuroimmunology, central nervous system, immune system, CNS repair, CNS disorders

The pathology of neurological disorders, such as multiple sclerosis (MS), Alzheimer's disease (AD), spinal cord injury, and stroke is characterized by immune responses located within the central nervous system (CNS). This close neuro-immune interaction is not only involved in the onset, progression, and clinical manifestation of neurological diseases, but in some cases also plays a protective or even regenerative role. A complex interplay between peripheral immune cells, cells of the neurovascular unit, and CNS resident immune and glial cells steers the destructive, as well as the regenerative capacity of the CNS. An additional layer of immunological complexity is provided by the interaction between the adaptive and innate immune system in both inflammation and repair. Elucidating the underlying mechanisms involved in the neuro-immune interplay, may provide deep insight into novel therapeutic leads for future treatment strategies.

This Research Topic provides a comprehensive overview of neuro-immunological connections that enable vs. hamper CNS repair. High-quality research papers, as well as detailed overview papers have been collected in five different sections.

First, molecular mechanisms involved in peripheral and/or central immune cell functioning related to CNS disorders and repair are addressed. Evans et al. and Yeola et al. describe peripheral T cell-mediated mechanisms in CNS disorders. Evans et al. provide an extensive overview of T cell subsets and mechanisms that are beneficial for neuro-inflammatory and neurodegenerative disorders. They describe recently discovered pro-regenerative T cell-mediated mechanisms in amyotrophic lateral sclerosis, AD, Parkinson's disease, MS, and CNS trauma/injury. Yeola et al. investigated cellular mechanisms in the pathogenesis of experimental autoimmune encephalomyelitis (EAE), the animal model for MS. They studied EAE in the 1C6 NOD model, where T cells have a transgenic T cell receptor (TCR) which is specific for MOG_35−55_. They found that regulatory T cells (Tregs) are essential for suppression of CNS autoimmunity in this model, and that endogenous TCR rearrangements, *via* recombination-activating gene (RAG) enzymes, are necessary for the development of these Tregs. With regard to central immune mechanisms, both Galloway et al. and Gervois and Lambrichts describe microglial functions, with a focus on phagocytosis, in health and disease. Gervois and Lambrichts provide an overview of TREM2- mediated microglial functions, including phagocytosis, migration, survival, and a pro-regenerative phenotype switch, which could be harnessed in the resolution of ischemic stroke. Galloway et al. provide an overview of the mechanisms of phagocytosis in the brain and how it is involved in brain injury and repair. They describe the role of phagocytosis in brain development, homeostasis and aging, and its function in disease states, including acute injury, MS, and AD.

Second, the blood brain barrier (BBB), which is the gateway to the brain, was addressed in this Research Topic. Wouters et al. found that liver X receptor (LXR) alpha, and not LXR-beta, is crucial for maintaining BBB integrity and immune quiescence, in a mouse model for MS. LXRs are ligand-activating transcription factors with important roles in cholesterol and lipid metabolism, but as this report now shows, they are also involved in neuro-inflammatory processes.

Third, the identification of novel molecular leads in the prevention and regeneration of neuro-immunological disorders is an important section of this Research Topic. Not only the identification and classification, but in particular the preclinical validation of potential targets provide key leads for future treatment strategies. In a first study, Sisa et al. show that properdin, a positive regulator of alternative complement activation, is crucially involved in neonatal hypoxia-ischemia induced brain damage. The results indicate that global properdin deletion in two independent mouse models for hypoxic ischemia (HI), reduced forebrain cell death, microglial activation, and tissue loss. The identification of properdin as a mediator of HI, renders properdin an interesting target to prevent HI-induced CNS damage. Next, Schepers et al. provide an elaborative overview on the involvement of second messengers in neuroinflammation and CNS repair. Intracellular second messengers are tightly regulated by phosphodiesterases (PDEs). The unique cell type-specific fingerprint of different PDE isoforms allows a tailor-made treatment strategy. Inhibition of selected PDEs in MS limits inflammation, while inhibition of others stimulates regenerative processes. Kolahdouzan et al. review novel therapies currently in clinical trial, and that are likely to appear in clinical practice in the near future. They focus on compounds that target the immune system and/or enhance endogenous repair mechanisms in the CNS. Although the authors primarily discussed the upcoming treatments in the context of MS, they indicate that most of the strategies can be extrapolated to the treatment of other neuro-inflammatory disorders.

Fourth, the contribution of glia to CNS repair was addressed, with a specific focus on the impact of immune mediators on glial function. Houben et al. provide a detailed overview of the known functions of oncostatin M (OSM), a neuropoietic cytokine, in CNS homeostasis and pathology. Here, they focus on the effects of OSM on neurons, astrocytes, microglia/macrophages and BBB endothelial cells, and discuss the current insights of OSM's involvement in reparative processes seen in murine models of CNS pathology. Lee et al. provide an overview of TNF superfamily reverse signaling in phagocytes of the CNS, both in physiological and pathological circumstances. In addition, they discuss the possibility of targeting these pathways for clinical application. Two reports from Kamermans, Rijnsburger et al. and Kamermans, Verhoeven et al. describe novel pathways showing how astrocytes are involved in MS pathogenesis. First, Kamermans, Verhoeven et al. showed that melanocortin receptor 4 (MC4R) is expressed on astrocytes in active MS lesions, and that activation of astrocytic MC4R ameliorates their reactive phenotype. These data suggest that targeting MC4R on astrocytes might be a novel therapeutic strategy to halt inflammation-associated neurodegeneration in MS. Second, Kamermans, Rijnsburger et al. showed that the expression of angiopoietin-like protein 4 (ANGPTL4), which is an inhibitor of lipoprotein lipase (LPL), is reduced on astrocytes in active MS lesions. ANGPTL4 inhibits uptake of myelin-derived lipids by LPL-expressing phagocytes. These data suggest that the strong reduction in astrocytic ANGPTL4 expression in active demyelinating MS lesions enables phagocytes to adequately clear myelin debris, setting the stage for remyelination.

Fifth and finally, other immunopathogenic mechanisms involved in neurodegeneration were addressed. For this part, four review papers discuss a diverse collection of CNS disorders and immunopathogenic mechanisms. Salani et al. summarize evidence evoking innate immune memory mechanisms in AD, and interpret their potential function, either protective or harmful, in disease progression. Mazón-Cabrera et al. provide an extensive overview of the antibodies described in autism spectrum disorders according to their target antigens, their different origins, and timing of exposure during neurodevelopment. Jin et al. review recent progress in understanding how BDNF influences mood disorders, by participating in alterations of the neuro-immune axis. Wang et al. highlight and discuss how the host microbiome, as a crucial extrinsic factor, influences microglia within the CNS. In addition, they summarize which CNS diseases are associated with host microbiome and microglia alterations and explore potential pathways by which gut bacteria can influence the pathogenesis. He et al. show that microglia mediate the remodeling of rod bipolar cells by phagocytosing postsynaptic materials and inhibiting ectopic neuritogenesis, thus reducing the deterioration of vision in a rat model of retinitis pigmentosa. Wetzels et al. show that advanced glycation end products (AGEs) are increased in MS brain lesions, and specifically expressed in astrocytes. Their receptors, RAGEs, are expressed on brain phagocytes, and together, this system could contribute to MS pathology.

In conclusion, this Research Topic emphasizes the importance of exploiting immunological mechanisms to boost repair in CNS disorders. In depth knowledge of these complex neuro-immune interactions will feed the pipeline of novel treatment paradigms to efficiently treat a variety of diseases, for which currently no optimal treatment options exist.

## Author Contributions

TV, JH, NH, and BB edited the Research Topic and wrote the Editorial. All authors contributed to the article and approved the submitted version.

## Conflict of Interest

The authors declare that the research was conducted in the absence of any commercial or financial relationships that could be construed as a potential conflict of interest.

